# Effects of exercise/physical activity on fear of movement in people with spine-related pain: a systematic review

**DOI:** 10.3389/fpsyg.2023.1213199

**Published:** 2023-07-27

**Authors:** Ferozkhan Jadhakhan, Raghip Sobeih, Deborah Falla

**Affiliations:** Centre of Precision Rehabilitation for Spinal Pain (CPR Spine), School of Sport, Exercise and Rehabilitation Sciences, College of Life and Environmental Sciences, University of Birmingham, Birmingham, United Kingdom

**Keywords:** exercise, kinesiophobia, low back pain, neck pain, chronic pain, fear of movement

## Abstract

**Background:**

Kinesiophobia (i.e., fear of movement) can be an important contributor for ongoing pain and disability in people with spine-related pain. It remains unclear whether physical activity interventions/exercise influence kinesiophobia in this population. A systematic review was therefore conducted to synthesize the available evidence on whether physical activity interventions/exercise influence kinesiophobia in people with chronic non-specific spine-related pain.

**Methods:**

The study protocol was registered prospectively with PROSPERO (CRD42021295755). The following databases were systematically searched from inception to 31 January 2022 and updated on 22 June 2023: PubMed, MEDLINE, Embase, CINAHL, Web of Science, PsycINFO, ZETOC, PROSPERO and Google Scholar. Inclusion criteria were randomized or non-randomized controlled studies investigating adults aged ≥18 years, reporting the effect of exercise or physical activity on kinesiophobia in individuals with chronic non-specific spine-related pain. Two reviewers independently extracted data and assessed the quality of the included studies. Bias was assessed using the Cochrane ROB2 tool and evidence certainty via Grading of Recommendations Assessment, Development and Evaluation (GRADE).

**Results:**

Seventeen studies from seven countries involving a total of 1,354 individuals were selected for inclusion. The majority of studies (*n* = 13) involved participants with chronic low back pain (LBP), and Pilates was the most common form of exercise evaluated. Most of the studies reported a positive direction of effect in favor of exercise reducing kinesiophobia when compared to a control group. There was moderate to high risk of bias among the studies and the overall certainty of the evidence was very low.

**Conclusion:**

This review supports the use of exercise for reducing kinesiophobia in people with chronic LBP albeit with very low certainty of evidence; Pilates (especially equipment-based) was shown to be effective as were strengthening training programmes. There was limited evidence available on the effects of exercise on kinesiophobia for people with chronic neck or thoracic pain and further research is required.

**Systematic review registration:**

https://www.crd.york.ac.uk/prospero/display_record.php?RecordID=295755

## Introduction

Spine-related pain is a common global health issue and will continue to be due to the predictable nature of the world's aging population (World Health Organisation, 2021). Low back pain (LBP) has been established as one of the leading causes of disability-adjusted life years in the world for all ages (Vos, 2020). In Europe, 15% of people suffering from LBP are absent from work for over 1 month, which accounts for half of the number of days from work lost (Bevan, 2012). In the UK, 60% of adults will experience LBP in their lifetime, with up to 4% of adults aged below 45 years and up to 7% of adults aged over 45 years being disabled by chronic LBP (National Institute for Health and Care Excellence, 2022). Neck pain is also highly prevalent; globally the age standardized point prevalence of neck pain per 100,000 population is 3551.1 (95% CI; 3139.5–3977.9), with the UK showing the highest increase (Safiri et al., 2020). Neck pain is common in all age groups (Hogg-Johnson et al., 2009), and it is correlated with increased risk of disability and reduced quality of life (Hey et al., 2021).

Kinesiophobia is a concept associated with fear of physical movement that is “debilitating” and “excessive”, which is expressed through fear-avoidance behaviors (Leeuw et al., 2007). For many people with spine-related pain, kinesiophobia acts as an initial protective mechanism that prevents the potential exacerbation of their condition (Trocoli and Botelho, 2016), but kinesiophobia can lead to the development of chronicity of pain and its maintenance (Vlaeyen et al., 1995; Vlaeyen and Linton, 2000). Physical activity limitations are self-imposed due to the fear of pain that may result from certain movements, causing people with chronic pain to be progressively less active, increasing the risk of physical disability (De Moraes et al., 2014). A systematic review by Luque-Suarez et al. (2019) provided strong evidence of higher levels of kinesiophobia being associated with greater levels of disability, pain intensity and a lower quality of life in people with chronic musculoskeletal pain. Early identification of kinesiophobia may be crucial in order to design treatment programmes that can target heightened kinesiophobia levels to reduce this barrier to rehabilitation (Varallo et al., 2020), and provide more effective treatment that is characterized by an improvement in physical function, reduction in pain intensity and an overall improvement in quality of life.

Previous research has investigated the association between kinesiophobia and physical activity, and the effect certain exercises have on fear of movement in people suffering from spine-related pain, and a variety of conclusions have been made. A study by Balci et al. (2020) found that both land and aquatic-based exercises have a beneficial effect on kinesiophobia in individuals with chronic LBP. Furthermore, a systematic review published in 2020 investigating the effects of Pilates found moderate evidence that Pilates effectively reduced kinesiophobia in people with chronic non-specific LBP when compared to no or minimal interventions (De Freitas et al., 2020). Additionally, a scoping review conducted by Bordeleau et al. (2022) treatments for kinesiophobia in people with chronic pain, found that The Tampa Scale of Kinesiophobia is the most widely used tool for assessing kinesiophobia. Physical exercise is the most frequently used strategy for managing irrational fear of movement. Interventions for kinesiophobia have mainly focused on musculoskeletal pain conditions, particularly low back pain and neck pain. On the other hand, a systematic review and meta-analysis by Hanel et al. (2020) found low to very low-quality evidence that fear-avoidance beliefs were reduced by exercise, but they also concluded that non-exercise interventions were equally as effective in achieving the same outcome. This review assessed exercise approaches as a whole, without differentiating between different forms of exercise. Collectively, the results of this work show that the effect of exercise/physical activity on fear of movement/kinesiophobia remains unclear and evidence on the effect of exercise/physical activity on kinesiophobia in people with spine-related pain is needed.

The aim of this systematic review was to investigate whether physical activity interventions/exercise influence the level of kinesiophobia in people with chronic non-specific spine-related pain. It was anticipated that the results of this review would provide evidence of the impact of specific forms of exercise/physical activity on kinesiophobia in people experiencing chronic non-specific spine-related pain which would facilitate clinical decision making when prescribing exercise for patients with chronic non-specific spine-related pain presenting with kinesiophobia.

## Methods

### Search strategy

A comprehensive search of PubMed, MEDLINE, Embase, CINAHL, Web of Science, PsycINFO, ZETOC, PROSPERO and the first 10 pages of Google Scholar was conducted from inception to 31 January 2022 and updated on 22 June 2023. A search strategy (Supplementary material 1) was developed using the following key words: physical activity, exercise, kinesiophobia, fear of movement, spinal pain, neck pain, low back pain and randomized controlled trial and adapted for each database. Additional filters/limits were added when searching for articles on the databases where they were available. The search strategy was developed by RS and FJ and iteration discussed with DF. The review protocol was registered in PROSPERO (International Prospective Register of Systematic Reviews) (registration number: CRD42021295755) and published (Jadhakhan et al., 2022). Assuming homogeneity between studies, we planned to conduct a random effect meta-analysis with and without low quality studies. This review is conducted and reported in accordance with the Preferred reporting System and Meta-Analysis (PRISMA) 2020 statement (Page et al., 2021) and conducted following the Cochrane Handbook for Systematic Review of Interventions (Higgins et al., 2021) (Supplementary material 2).

### Inclusion and exclusion criteria

Studies were included if they were randomized or non-randomized controlled trials that investigated exercise or physical activity interventions for people with non-specific spine-related pain, with recorded measures of kinesiophobia taken at baseline and post-intervention. The inclusion and exclusion criteria of this review was determined using the Population, Interventions, Comparators, Outcomes and Study design (PICOS) framework (Richardson et al., 1995; Akers et al., 2009).

#### Population

Adults (≥ 18 years) with chronic non-specific spine-related pain (i.e., neck pain, thoracic pain and LBP).

#### Intervention

Any form of exercise/physical activity that may be deemed as structured with the objective of improving one or more aspects of an individual's physical fitness (Caspersen et al., 1985); interventions include: aerobic/cardiovascular exercise, Pilates, resistance/strength training, yoga, hydrotherapy, motor-control exercise, walking, core stabilization exercise.

#### Comparator

Any study that compared exercise/physical activity with a control group (e.g., usual care or waiting list) or passive interventions (such as education or manual therapy) or general practitioner management for kinesiophobia.

#### Outcome measures

Eligible studies must have reported kinesiophobia using validated measures. The Tampa Scale of Kinesiophobia (TSK) (Miller et al., 1991; Hudes, 2011), and the Fear Avoidance Beliefs Questionnaire (FABQ) (Waddell et al., 1993) are two of the most common validated measures for kinesiophobia (Sharma et al., 2020). Studies that used these were eligible. Studies that used any other validated measures for kinesiophobia were also eligible, such as the other commonly used Kinesiophobia Causes Scale (KCS), Fear-Avoidance Components Scale (FACS) and the Athletes Fear-Avoidance Questionnaire (AFAQ) (Liu et al., 2021).

#### Study design

Randomized controlled trials or non-randomized controlled trials.

#### Exclusion criteria

Studies not written in English were excluded, as were review articles, case reports, letters, editorials, single case studies, abstract, un-published work in non-peer reviewed journals and non-experimental study designs were also not considered. Studies which only included participants with a specific pathology for their pain, radiculopathy, a traumatic injury or if they were post-surgery patients.

### Study selection

**Figure 1:**
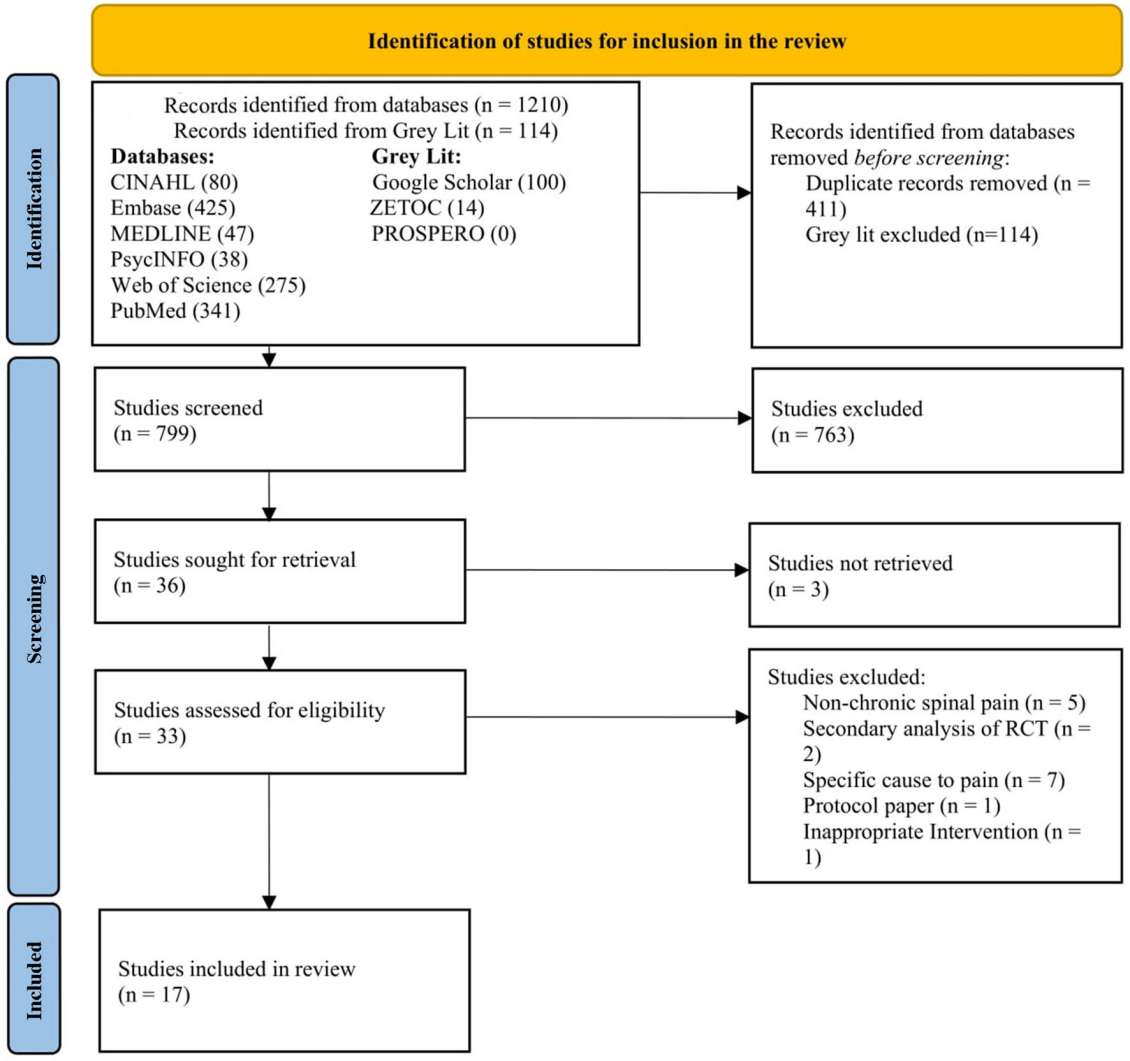
PRISMA flowchart for study screening (Page et al., 2021).

Screening results from the database searches were exported into a digital library using the Endnote version 20 reference management software (Clarivate Analytics, Philadelphia, PA, US). Duplicate records were automatically and manually removed. Within the digital library, the titles and abstracts of the articles were reviewed by two reviewers (RS and FJ) independently. Both reviewers discussed the included studies along with studies that required discussion for potential inclusion. Studies comparing two or more eligible exercise interventions were discussed between RS and FJ for inclusion only if the effects of the interventions were separately measured. In the event of disagreement between the two reviewers, a third reviewer (DF) adjudicated the eligibility of the article. In the event of full text availability for some of the selected studies, the lead author was contacted via email twice with a follow-up email sent 2 weeks apart. For the selected articles, full texts were acquired and screened using the same process with both reviewers (RS and FJ) to see if they met the inclusion criteria. A PRISMA (Page et al., 2021) flow diagram describes the process of the inclusion and exclusion of studies and reasons for exclusion from the latest screening stage (Figure 1).

### Data extraction

The data from each study was extracted by both RS and FJ and organized into a pre-determined data extraction sheet. Data items extracted from the eligible studies were: authors, publication year, title of the study and study design, country/setting, the characteristics of the participants (age, gender, ethnicity, spinal pain diagnosis, length of diagnosis), sample size, duration/frequency of interventions, length follow-ups, outcome measure, statistical methods, results and findings. Any discrepancies were resolved by discussion and re-visiting the relevant study. A third reviewer (DF) was available to mediate any disagreement in data extraction. If any information was missing or incomplete, an initial attempt was made to contact the study authors and a follow-up email sent 2 weeks after to retrieve the missing data. Descriptive data was extracted from the included studies and were summarized in a Microsoft Excel spreadsheet (Supplementary material 3).

### Risk of bias in individual studies

Risk of bias was assessed using Version 2 of the Cochrane risk-of-bias tool for randomized trials (RoB 2) (Sterne et al., 2019). The RoB-2 tool consists of five domains of bias: bias arising from the randomization process, bias due to deviations from intended interventions, bias due to missing outcome data, bias in the measurement of the outcome, and bias in the selection of the reported result. The Risk Of Bias in Non-randomized Studies of Interventions tool was planned to be used to assess the risk of bias of non-randomized studies of interventions however all included studies were randomized trials. Two reviewers (RS and FJ) independently assessed each of the included studies. A third assessor (DF) was available if needed. The reviewers (RS and FJ) used the RoB 2 Cribsheet to follow the algorithm to determine assessment of bias for the individual domains and then present the overall judgement of the study as either low risk, some concerns, or a high risk of bias.

### Evaluation of the certainty of evidence

After the evidence was collected and summarized, the assessment of certainty in the body of evidence was conducted in accordance with the Grading of Recommendations Assessment, Development and Evaluation (GRADE) guidelines (Guyatt et al., 2011) and performed by one person (FJ) using the GRADE rating guidance presented in the Cochrane Handbook (Balshem et al., 2011; Schünemann et al., 2021). Consistent with GRADE, the quality of the summary evidence was assessed as high, moderate, low or very low. For each study, the following domains were assessed: imprecision, inconsistency, indirectness, risk of bias including publication bias. Applicability of results were categorized by the study interventions and rated when making judgement about the quality of evidence presented in the included studies (Guyatt et al., 2011).

### Data analysis

Given the significant clinical and statistical variation between the studies included in this review, it was not possible to pool effect estimates of exercise/physical activity on kinesiophobia using meta-analysis. Variations was reported in extracted effect measures, population and measures used to ascertain kinesiophobia (e.g., TSK and FABQ). There was also difference among studies in sample size, interventions (including their duration and frequencies) length of follow-ups. Instead, we summarized effect estimates (mean difference or standardized mean difference) with 95% confidence intervals (CI) where appropriate in the included studies. Standardized Mean Difference (SMD) was extrapolated from reported (Cohen's *d*) values, exploring mean difference between groups; an effect size of 0.8 or greater was considered a relatively large effect size between two means in the sample population. In the event of missing data we attempted to contact the author(s) at least twice by email. A final reminder was sent to corresponding authors/co-authors if no response was received following our initial email. We initially planned to quantify heterogeneity using the Cochrane Q-test and the *I*^2^ with corresponding 95% (CI) where appropriate. Higher *I*^2^ values (>50%) (Higgins et al., 2021) indicate larger degrees of heterogeneity pertaining to variability in effect size estimates between studies (Higgins et al., 2021). Recorded measures of kinesiophobia in individuals aged (≥18 years) with chronic non-specific spine-related pain (i.e., neck pain, LBP, and thoracic pain) were extracted from each study and a narrative summary of the outcome of the included studies was presented.

### Grouping studies for synthesis

Due to different types of spine-related pain reported, studies were grouped by the type of spine-related pain (neck pain, thoracic pain and LBP) for synthesis rather than pooling across all spine-related pain.

## Results

In total, the search strategy yielded 1,324 articles. After excluding 411 duplicates and 114 gray literatures, the titles and abstracts of 795 articles were screened for relevance. Title and abstract screening resulted in the exclusion of 766 articles, primarily because these articles included participants with non-chronic spinal pain, conducted secondary data analysis, included participants with specific cause of pain, or used other types of intervention. Of the 33 full-text articles that were assessed, 16 were excluded after further review. Five studies were excluded because only participants with non-chronic spine-related pain were included, another two studies used secondary data from existing RCT's, seven reported specific cause of pain, one used other type of intervention and one study was a trial protocol. Seventeen articles were included in the final analysis. A flow diagram of the study selection process is presented in Figure 1.

### Study characteristics

The characteristics of the included studies are presented in Table 1. The 17 studies selected were published between the years 2011 to 2023. The 17 selected studies (Nassif et al., 2011; Miyamoto et al., 2013, 2018; Da Luz Junior et al., 2014; Vincent et al., 2014; Cruz-Díaz et al., 2017, 2018; Keane, 2017; Zadro et al., 2019; Galan-Martin et al., 2020; Tagliaferri et al., 2020; Akodu et al., 2021; Vicente-Campos et al., 2021; Martins de Sousa et al., 2022; Cana-Pino et al., 2023; Hernandez-Lucas et al., 2023; Ogunniran et al., 2023) involved 1,354 participants. Variation in chronic non-specific spine-related pain, exercise/physical activity and measures used to assess kinesiophobia contributed to the significant level of heterogeneity between the studies and reported effect estimates. Most studies were performed in Spain (*n* = 6), followed by Brazil (*n* = 4), Australia (*n* = 2), Nigeria (*n* = 2), England (*n* = 1), France (*n* = 1) and the United States (*n* = 1). Most studies were conducted in a community clinic setting and University Laboratory (*n* = 8), followed by outpatient units (*n* = 3), primary care centers (*n* = 2), physical activity training unit (*n* = 2), private clinic (*n* = 1) and participant homes (*n* = 1). Studies included in this review were mostly randomized controlled trials (*n* = 9), followed by single blinded RCTs (*n* = 6), double blinded RCT (*n* = 1) and one repeated measures RCT (*n* = 1). Across all the studies, 64% (*n* = 871) of participants were female and 36% (*n* = 483) were male. The ethnicity of the participants was not reported in any of the studies. Thirteen studies recruited people with chronic LBP, three studies recruited people with chronic neck pain, and one study recruited people with (any sort of) chronic spine-related pain. The length of follow-up across the studies were between 4 weeks and 12 months. Frequency of exercise/physical activity was between 30 min and 1 h per session.

**Table 1:** Demographic data for included studies.

**Study details**				**Demographic information**				
**Authors (year)**	**Country**	**Study design**	**Setting**	**Age**	**Gender**	**Spinal pain diagnosis criteria**	**Length of diagnosis**	**Sample size**
Akodu et al. (2021)	Nigeria	Single Blinded RCT	Outpatients - Hospitals in the Lagos state	I (Pilates only) = 47.43 ± 9.22. I (Neck stabilization group) = 47.71 ± 10.02 C = 44.93 ± 6.26	Male = 19 (42%) Female = 26 (58%)	Non-specific Chronic neck pain ≥ 3 months. Participants with neck pain greater or equal to 5/10	≥ 3 months	45
Cruz-Díaz et al. (2017)	Spain	RCT	Physical therapy unit - Jaén	I (Mat Pilates) = 36.94 ± 12.46 I (Equipment based Pilates) = 35.5 ± 11.98 C = 36.32 ± 10.67	Male = 35 (36%) Female = 63 (64%)	History of LBP ≥ 12 weeks; pain between 3 and 10 on 10 cm VAS	≥ 12 weeks	98
Cruz-Díaz et al. (2018)	Spain	Single Blinded RCT	Physiotherapy laboratory of University of Jaén	I (Mat Pilates) = 36.94 ± 12.46 I (Equipment based Pilates) = 35.5 ± 11.98 C = 36.32 ± 10.67	64 total, 2 excluded. Male = 21 (34%) Female = 41 (66%)	LBP ≥ 3 months	≥ 3 months	64 (62 as 2 excluded)
Da Luz Junior et al. (2014)	Brazil	RCT	Private physiotherapy clinic	I (Mat Pilates) = 43.5 ± 8.6 I (Equipment based Pilates) = 38.8 ± 9.9	Male = 20 (23%) Female = 66 (77%)	LBP > 3 months	> 3 months	86
Galan-Martin et al. (2020)	Spain	RCT	12 Primary Care centers in Valladolid	I = 53.02 ± 10.7 C = 49.14 ± 12.14	Male = 34 (20%) Female = 136 (80%)	Non-specific CSP > 6 months	> 6 months	170
Keane (2017)	England	Repeated measures RCT	Aspire National Training Center	46 ± 17	Male = 5 (17%) Female = 24 (83%)	CLBP ≥ 3 months	≥ 3 months	29
Miyamoto et al. (2013)	Brazil	RCT	outpatient physical therapy department	I = 40.7 ± 11.8 C = 38.3 ± 11.4	Male = 16 (19%) Female = 70 (81%)	Non-specific LBP ≥ 3 months	≥ 3 months	86
Miyamoto et al. (2018)	Brazil	RCT	Physiotherapy clinic - Sao Paulo	I (P1) = 47.0 ± 11.5 I (P2) = 47.1 ± 14.9 I (P3) = 48.9 ± 16.6 C = 48.6 ± 15.8	Male = 72 (24%) Female = 224 (76%)	Non-specific CLBP > 3 months	> 3 months	296
Nassif et al. (2011)	France	RCT	Workplace of French automotive manufacturer (Peugeot Citroen, Mulhouse)	I = 45.13 ± 9.11 C = 45.34 ± 8.80	Male = 43 (57%) Female = 32 (43%)	Chronic LBP	Not stated	75
Tagliaferri et al. (2020)	Australia	RCT	Clinical exercise and healthcare centers	I (GSC) = 34.8 ± 4.9 I (MCMT) = 34.6 ± 7.2	Male = 21 (52.5%) Female = 19 (47.5%)	Non-specific CLBP > 3 months	> 3 months	40
Vicente-Campos et al. (2021)	Spain	Single blinded RCT	Not reported (?assumed at Francisco de Vitoria University)	I = 23.25 ± 4.52 C = 23.90 ± 7.36	Male = 16 (40%) Female = 24 (60%)	Non-specific CLBP	At least 3 episodes in last 6 months	40
Vincent et al. (2014)	USA	RCT	Laboratory	I (LEXT) = 68.7 ± 7.1 I (TOTRX) = 68.6 ± 7.1 C = 67.5 ± 6.4	Men: I (LEXT) = 32%. I (TOTRX) = 29.2% C = 38.9%	LBP ≥ 6 months	≥ 6 months	49
Zadro et al. (2019)	Australia	Single blinded RCT	Participant's homes	68.3 ± 5.7	Male = 29 (48.3%) Female = 31 (51.7%)	Non-specific mechanical LBP ≥ 3 months	≥ 3 months	60
Cana-Pino et al. (2023)	Spain	Single blinded RCT	Private physiotherapy clinic	I - supervised exercise (SE) + pain neuroscience education (PNE) - 35.3+/−7.10 - I - Laser guided exercise + pain neuroscience education (PNE) - 32.0+/−6.78	Not reported	Non-specific CLBP ≥ 3 months	≥3 months	60
Ogunniran et al. (2023)	Nigeria	Single blinded RCT	from the physiotherapy outpatient clinics of both tertiary and secondary health hospitals	Kinesiology taping + core stabilization exercises = 42.1+/−12.0; Core stabilization = 42.3+/−10.8; kinesiology taping = 43.7+/−9.5	Male = 29 (67.4%); Female = 7 (16.3%)	Non-specific CLBP 2 months	2 months	43
Martins de Sousa et al. (2022)	Brazil	Double blind randomized controlled trial	Physical Education Department of Universidade Federal do Maranhão, Brazil	Control (group 1) = 30.40 (+/−7.74); intervention (group 2) = 29.35 (+/−8.80); intervention (group 3) = 31.55 (+/−6.13)	Control (group 1) = F:12; M:8; GROUP 2: F = 15; M = 5; GROUP 3: F = 14; M = 6	Non-specific chronic neck pain for more than 3 months	≥3 months	60
Hernandez-Lucas et al. (2023)	Spain	Randomized controlled clinical trial	The Pontevedra Sport Center (Spain)	Intervention: People attending the Back- School Program (BSP) = 51.0+/−7.6 - Control group = People who did not attend the BSP = 50.7+/−10	EG: F = 18; M = 10 ; CG: F = 17; M = 10	“Non-specific neck pain for at least three months, with pain intensity of 30–70 on the visual analog scale (VAS).”	3 months	55

I, Intervention group; C, Control group; P, Pilates; LBP, Low Back Pain; CLBP, Chronic Low Back Pain; CSP, Chronic Spinal Pain; RCT, Randomized controlled trial; LEXT – Lumbar Extension Resistance Training; TOTRX – Total Body Resistance Training; GSC, General strength and conditioning training; MCMT, Motor Control and Manual Therapy.

### Risk of bias assessment

Key features affecting the methodological quality of each reviewed study are presented in Table 2. There was significant risk of bias detected across the studies, with the overall risk of bias considered high or with some concerns of bias. This was largely because of blinding, allocation sequence concealment and deviation from intended intervention. Five studies (Nassif et al., 2011; Miyamoto et al., 2013, 2018; Zadro et al., 2019; Galan-Martin et al., 2020) were rated as having high risk of risk, ten (Vincent et al., 2014; Keane, 2017; Cruz-Díaz et al., 2018; Tagliaferri et al., 2020; Akodu et al., 2021; Vicente-Campos et al., 2021; Martins de Sousa et al., 2022; Cana-Pino et al., 2023; Hernandez-Lucas et al., 2023; Ogunniran et al., 2023) with some concerns of bias and two (Da Luz Junior et al., 2014; Cruz-Díaz et al., 2017) deemed low risk. Most studies adequately described the follow-up period, but attrition rate was poorly defined. Only two studies had a low risk of bias in all criteria of the checklist (Da Luz Junior et al., 2014; Cruz-Díaz et al., 2017). For all studies, the analytical approach utilized was considered appropriate. In seven studies (Vincent et al., 2014; Keane, 2017; Tagliaferri et al., 2020; Akodu et al., 2021; Vicente-Campos et al., 2021; Hernandez-Lucas et al., 2023; Ogunniran et al., 2023) consisted of a small sample size which is a major limitation and demonstrate a lack of adequate sample size calculation.

**Table 2:** Risk of Bias-2 (RoB-2) Assessment of included studies.

	**Akodu et al. (2021)**	**Cruz-Díaz et al. (2017)**	**Cruz-Díaz et al. (2018)**	**Da Luz Junior et al. (2014)**	**Galan-Martin et al. (2020)**	**(Keane, 2017)**	**Miyamoto et al. (2013)**	**Miyamoto et al. (2018)**	**Nassif et al. (2011)**	**Tagliaferri et al. (2020)**	**Vicente-Campos et al. (2021)**	**Vincent et al. (2014)**	**Zadro et al. (2019)**	**Cana-Pino et al. (2023)**	**Ogunniran et al. (2023)**	**Hernandez-Lucas et al. (2023)**	**Martins de Sousa et al. (2022)**
Domain 1																	
Domain 2																	
Domain 3																	
Domain 4																	
Domain 5																	
Risk of Bias																	
																	Low risk
																	Some concerns of bias
																	High risk

Domain 1: Risk of bias arising from the randomization process. Domain 2: Risk of bias due to deviations from the intended interventions (effect of assignment/adhering to intervention). Domain 3: Missing outcome data. Domain 4: Risk of bias in measurement of the outcome. Domain 5: Risk of bias in the selection of the reported results.

### Certainty of evidence

For all 17 studies, the result of the GRADE was very low suggesting the true effect is likely to be substantially different from the estimated effect (Schünemann et al., 2021) (Table 3), thus suggesting very little confidence in the effect estimates. Three studies (Da Luz Junior et al., 2014; Cruz-Díaz et al., 2017, 2018) used two types of exercise intervention (Mat and equipment based Pilates) and a GRADE ranking was undertaken. The GRADE ranking indicates low certainty that the Pilates interventions are effective at reducing fear of movement with relatively small sample size RCT's indicating larger fully-powered trials are needed to adequately test this assumption. Another study (Akodu et al., 2021) investigated the effect of neck stabilization and Pilates exercise on kinesiophobia measured by TSK. The GRADE ranking indicated low certainty that there is no effect on kinesiophobia following Pilates and neck stabilization exercise. Two studies (Miyamoto et al., 2013, 2018) investigated the effect of the frequency of Pilates and education on fear of movement measured by TSK; the GRADE ranking was very low for both interventions, meaning that we have little certainty that the observed effects are the true effects of these interventions. Two further studies (Keane, 2017; Galan-Martin et al., 2020) tested the effectiveness of land based stretching and Aqua stretch exercise and these were rated as very low. Four studies (Nassif et al., 2011; Vincent et al., 2014; Tagliaferri et al., 2020; Vicente-Campos et al., 2021) examined the effect of general strength and conditioning, muscle strengthening, motor control exercise with manual therapy Hypopressive Abdominal Gymnastics and Lumbar extension resistance exercise on fear of movement measured by either the TSK, TSK-11 (Spanish version) and FABQ; the GRADE rating was either low or very low, suggesting little evidence that the observed effects are the true effects of these interventions. Another study (Zadro et al., 2019) assessed the effect of video game-based exercise (Wii Fit U) on fear of movement measured by TSK; the GRADE rating was also very low. Another study (Cana-Pino et al., 2023) examined the effect of supervised/laser guided exercise and neuroscience education (PNE) on fear of movement measured by TSK-11 (Spanish version), the GRADE ranking was very low suggesting little evidence that the observed effects are the true effects of these interventions. Ogunniran et al. (2023) examined the effects of kinesiology taping and core stability exercise on clinical variables in patients with non-specific chronic low back pain. Fear of movement/Kinesiophobia was measured by TSK, the GRADE rating was very low. Another study (Hernandez-Lucas et al., 2023) tested the effects of the Back School-based intervention on non-specific neck pain in adults. The Back schools are an educational and exercise programs with lessons given to patients or workers by a therapist with the aim of treating or preventing low back pain. Fear of movement/Kinesiophobia was measured by TSK-11 (Spanish version). The GRADE ranking indicated low certainty that there is no effect on treating/preventing low back pain following the Back School intervention. Additionally, in a study (Martins de Sousa et al., 2022) evaluating the effect of high frequency (HF) or low frequency (LF) transcutaneous electrical nerve stimulation (TENS) in a specific therapeutic exercise program for the treatment of patients with chronic neck pain; were rated as low. Fear of movement/Kinesiophobia - measured by TSK.

**Table 3:** GRADE rating on the level of evidence of the included studies.

	**Quality assessment**							**Number of patients**		**Quality**

**Intervention**	**No of studies**	**Design**	**Risk of Bias**	**Inconsistency**	**Indirectness**	**Imprecision**	**Publication bias**	**Intervention**	**Comparison**	
Mat PILATES AND EQUIPMENT BASED PILATES	3	RCT	Serious limitations	No serious inconsistency	Serious indirectness	Serious imprecision	Likely	138	105	Very low
Pilates and Neck stabilization	1	RCT	Serious limitations	No serious inconsistency	Serious indirectness	Serious imprecision	Likely	31	14	Very low
Pain neuroscience education and group physical exercise	1	RCT	Serious limitations	No serious inconsistency	No serious indirectness	Serious imprecision	Likely	89	81	Very low
Land based stretching and Aqua stretch	1	RCT	Serious limitations	No serious inconsistency	Serious indirectness	Serious imprecision	Likely	20	9	Very low
Exercise based Pilates and education	1	RCT	Serious limitations	No serious inconsistency	Serious indirectness	Serious imprecision	Likely	43	43	Very low
Frequency of Pilates (1,2 and 3 times a week)	1	RCT	Serious limitations	Serious inconsistency	Serious indirectness	Serious imprecision	Likely	222	74	Very low
Muscle strengthening, flexibility and endurance training	1	RCT	Serious limitations	No serious inconsistency	Serious indirectness	Serious imprecision	Likely	37	38	Very low
General strength and conditioning v/s motor control and manual therapy	1	RCT	Agree with the criterion	No serious inconsistency	Mostly disagree with the criterion	Serious imprecision	Likely	20	20	Low
Hypopressive abdominal gymnastics	1	RCT	Serious limitations	No serious inconsistency	Serious indirectness	Serious imprecision	Likely	20	20	Very low
Total body resistance exercise and lumbar extensor exercise	1	RCT	Serious limitations	No serious inconsistency	Serious indirectness	Serious imprecision	Likely	42	18	Very low
Home-based Wii Fit U flexibility, strengthening, and aerobic exercises	1	RCT	Serious limitation	Serious inconsistency	Serious indirectness	Serious imprecision	Likely	30	30	Very low
Kinesiology taping and core stabilization exercises	1	RCT	Serious limitations	Serious inconsistency	Serious indirectness	Serious imprecision	Likely	30	13	Very low
Supervised/laser guided exercises and pain neuroscience education	1	RCT	Serious limitations	Serious inconsistency	Serious indirectness	Serious imprecision	Likely	30	30	Very low
People attending the back school program	1	RCT	Agree with the criterion	No serious inconsistency	Mostly disagree with the criterion	Serious imprecision	Likely	29	29	Low
Therapeutic exercise and high/low TENS	1	RCT	Serious limitations	Serious inconsistency	Serious indirectness	Serious imprecision	Likely	40	20	Very low
Outcome: TSK, FABQ, KCS, FACS, AFAQ scores

TSK, Tampa Scale of Kinesiophobia; FABQ, Fear Avoidance Beliefs Questionnaire; KCS, Kinesiophobia Causes Scale; FACS, Fear Avoidance Components Scale; AFAQ, Athletes Fear Avoidance Questionnaire.

### Characteristics of the included trials

#### Type of intervention

We noted considerable variation in the exercise intervention across studies. The exercise interventions among all the studies included Pilates, strengthening/stabilization education/exercise programmes (general and neck/lumbar specific), kinesiology taping and core stabilization exercise, supervised/laser guided exercises- and pain neuroscience education and forms of stretching. Some studies (Miyamoto et al., 2013; Zadro et al., 2019; Galan-Martin et al., 2020; Tagliaferri et al., 2020; Cana-Pino et al., 2023; Hernandez-Lucas et al., 2023) combined their interventions with aerobic exercise, supervised exercise and education such as pain neuroscience education. Other studies (Martins de Sousa et al., 2022; Ogunniran et al., 2023) used a combination of High- and low-frequency transcutaneous electrical nerve stimulation and exercise and kinesiology taping and core stabilization exercise. The control groups among the studies (Nassif et al., 2011; Miyamoto et al., 2013, 2018; Vincent et al., 2014; Cruz-Díaz et al., 2017, 2018; Keane, 2017; Zadro et al., 2019; Galan-Martin et al., 2020; Akodu et al., 2021; Vicente-Campos et al., 2021; Cana-Pino et al., 2023) either had no intervention, had usual physiotherapy treatment, or a form of education. From all the studies in the review, 14 (Nassif et al., 2011; Miyamoto et al., 2013, 2018; Vincent et al., 2014; Cruz-Díaz et al., 2017, 2018; Keane, 2017; Zadro et al., 2019; Galan-Martin et al., 2020; Akodu et al., 2021; Vicente-Campos et al., 2021; Martins de Sousa et al., 2022; Hernandez-Lucas et al., 2023; Ogunniran et al., 2023) had a control group, and ten studies (Da Luz Junior et al., 2014; Vincent et al., 2014; Cruz-Díaz et al., 2017; Keane, 2017; Miyamoto et al., 2018; Tagliaferri et al., 2020; Akodu et al., 2021; Martins de Sousa et al., 2022; Cana-Pino et al., 2023; Ogunniran et al., 2023) compared two or more interventions.

#### Duration of the intervention

The duration of the interventions ranged from 4 weeks to 6 months. Three studies (Miyamoto et al., 2013, 2018; Da Luz Junior et al., 2014) had a 6 weeks intervention, 5 studies (Zadro et al., 2019; Akodu et al., 2021; Vicente-Campos et al., 2021; Hernandez-Lucas et al., 2023; Ogunniran et al., 2023) had an eight-week intervention duration. Another three studies (Cruz-Díaz et al., 2017, 2018; Keane, 2017) applied the intervention for 12 weeks. The remaining 6 studies (Nassif et al., 2011; Vincent et al., 2014; Galan-Martin et al., 2020; Tagliaferri et al., 2020; Martins de Sousa et al., 2022; Cana-Pino et al., 2023) had an intervention duration of 20 weeks, 11 weeks, 4 weeks, 2 months, 6 months or 4 months.

#### Length of follow-up

Four studies had follow-ups at 6 and 12 weeks following the end of the intervention period (Cruz-Díaz et al., 2017, 2018; Keane, 2017; Cana-Pino et al., 2023). Three studies (Miyamoto et al., 2013, 2018; Da Luz Junior et al., 2014) had follow-up at 6 weeks, 6 and 12 months. Another five studies (Zadro et al., 2019; Akodu et al., 2021; Vicente-Campos et al., 2021; Martins de Sousa et al., 2022; Ogunniran et al., 2023) had follow-ups at 4 and 8 weeks. A further three studies (Nassif et al., 2011; Tagliaferri et al., 2020; Cana-Pino et al., 2023) had follow-ups at 2, 3 and 6 months. One study had a follow-up at the end of a 4-week intervention (Vincent et al., 2014). Another study (Hernandez-Lucas et al., 2023) did not report follow-up period.

#### Outcome measures characteristics

We noted significant diversity in the criteria utilized to ascertain the presence of kinesiophobia. The most important differences were: (1) where studies used different versions of the self-assessment questionnaire (TSK) or FABQ to assess kinesiophobia. Eleven studies (Nassif et al., 2011; Miyamoto et al., 2013, 2018; Da Luz Junior et al., 2014; Cruz-Díaz et al., 2017; Keane, 2017; Zadro et al., 2019; Tagliaferri et al., 2020; Akodu et al., 2021; Martins de Sousa et al., 2022; Ogunniran et al., 2023) used the 17 items TSK questionnaire. One study (Vincent et al., 2014) used a combination of FABQ and the shorthand TSK-11 version to assess fear of movement/kinesiophobia. Five studies (Cruz-Díaz et al., 2018; Galan-Martin et al., 2020; Vicente-Campos et al., 2021; Cana-Pino et al., 2023; Hernandez-Lucas et al., 2023) used the Spanish version of the shorthand TSK-11 to measure fear of movement/kinesiophobia.

### Effect of exercise on kinesiophobia

Findings from the included studies have been pooled to describe the effect exercise has on kinesiophobia for people with different types of spine-related pain.

### Chronic neck pain

One study (Akodu et al., 2021) investigated the effect of neck stabilization exercise and Pilates on kinesiophobia and found that both neck stabilization exercise (*Z* = −3.077; *p* = 0.002) and Pilates (*Z* = −2.994; *p* = 0.003) significantly reduced kinesiophobia levels at 8 weeks following the intervention compared to dynamic isometric neck exercises over the same period. This study indicates Pilates and neck stabilization exercises are effective in reducing kinesiophobia over 8 weeks in people with chronic neck pain. Another study (Martins de Sousa et al., 2022) evaluating the effects of high and low frequency transcutaneous electrical nerve stimulation (TENS) and a series of therapeutic exercises which include performing flexion, extension, inclination, and rotation movements of the cervical spine for the treatment of patients with chronic neck pain. The study found no significant clinical difference between participants allocated to the therapeutic exercise and placebo-TENS (group 1) and participants in the low TENS group (group 2) and kinesiophobia [mean difference between the groups−0.40 (−4.64, 3.84), *p* > 0.05], mean difference placebo TENS and high TENS was also not significant [mean difference between the groups −0.31 (−5.68, 5.06), *p* > 0.05]. The Back schools-based intervention, an educational and training programs with lessons given to patients or workers by a therapist with the aim of treating or preventing back pain was used by Hernandez-Lucas et al. (2023). This study investigated the effects of a Back schools-based intervention on non-specific cervical pain in an adult population. The study found a significant treatment effect between the experimental group (people attending the Back school program) compared to control (People who did not attend the Back school program) on TSK-11; [mean difference 7.0 (95% CI: −8.3 to −5.4), *p* < 0.001, *g* = 2.04].

### Chronic low back pain

Pilates was an intervention for people with chronic LBP in five studies, with four of them showing beneficial effects in reducing kinesiophobia. One study found that both mat Pilates and equipment-based Pilates significantly reduced kinesiophobia over 6 weeks and 12-week intervention period, and both differences were significant (*p* < 0.05) when compared to the control group (Cruz-Díaz et al., 2017). A significant reduction in TSK scores were observed at 12 weeks following Mat Pilates, with mean scores of 31.7 (+/−3.2) at 12 weeks compared to 34.5 (+/−4.1) at baseline. Equipment based Pilates also showed a significant improvement in TSK scores, with mean scores of 32.0 (+/−3.6) at 12 weeks compared to 36.5 (+/−3.9) at baseline. In contrast Da Luz Junior et al. (2014) compared a six-week equipment-based and mat Pilates interventions and found that after 6 month there was a statistically significant improvement in kinesiophobia [mean difference = 4.9 points (95% CI 1.6 to 8.2)] following equipment-based Pilates. These results suggests that equipment-based Pilates may have a longer-term effect on kinesiophobia levels than mat Pilates.

A study (Miyamoto et al., 2018) investigating the frequency of Pilates administered (once, twice and three times per week) in three groups of patients compared to advice/education, found that Pilates significantly reduced kinesiophobia at a 6 weeks follow-up. Participants having Pilates twice a week showed the greatest reduction in kinesiophobia, baseline mean 40.8 (+/−7.5) compared to 37.4 (+/−8.7) at 6 weeks. However, no significant difference was found for any of the three groups at 6- and 12-months follow-up. Additionally, Cruz-Díaz et al. (2018) found that after a 12-weeks Pilates exercise programme, the exercise group showed a significant improvement in kinesiophobia when compared to the control group at six [mean change 5.5 (+/−0.7), *p* < 0.001] and 12 weeks follow-up [mean change 5.0 (+/−0.8), *p* < 0.001]. Conversely, another study (Miyamoto et al., 2013) found that Pilates combined with an educational booklet resulted in no significant between group differences (booklet group v/s Pilates) at a 6 months follow-up [adjusted mean difference 0.6 (95% CI: −1.8 to 3.1), *p* = 0.61].

Keane (2017) assessed the effect of land and water-based stretching compared to a control group in people with chronic LBP and found that after 12 weeks, only water-based stretching significantly (baseline mea*n* = 37.1 v/s 12 weeks mean = 28.8, *p* = 0.03) reduced kinesiophobia. Nassif et al. (2011) investigated the effect of major muscle group training in people with LBP compared to a control group that had no direct intervention. Strengthening exercises significantly improved kinesiophobia post intervention [baseline mean 46.7 (+/−6.8)] at 2 months follow up [mean 41.6 (+/−6.93), *p* < 0.001]. Additionally, a general strength conditioning exercise programme reduced kinesiophobia [mean difference −6.6 (−9.9, −3.2, *p* < 0.001)] compared to a motor control (combined with manual therapy) exercise programme at the 6 months follow-up (Tagliaferri et al., 2020). On the other hand, Vicente-Campos et al. (2021) found no significant improvement in kinesiophobia following Hypopressive Abdominal Gymnastics programme compared to a control group [mean difference −2.00 (95% CI: −4.75 to −0.75, *p* = 0.15] after 8 weeks. Furthermore, in a study of obese older adults with chronic LBP, the effect of two exercise protocols [total body resistance training (TOTRX) and a lumbar extension resistance exercise training (LEXT)] were compared to a control group (standard care) for 4 months on fear of movement or re-injury and avoidance behavior. At the four-month follow-up, there was no statistical difference between the two exercise groups: TSK: TOTRX: mean baseline 24.5 (+/6.6) at 4 months mean 21.0 (+/−6.9); LEXT mean baseline 25.2 (+/−6.7) at 4 months mean 20.9 (+/−5.9). FABQ: TOTRX: mean baseline 13.2 (+/−14.2) at 4 months mean 8.3 (+/−10.5); LEXT mean baseline 11 (+/−5.9) at 4 months 9.1 (+/−7.2). Both exercise modalities shows improvement in fear of movement and avoidance behavior Vincent et al. (2014). Zadro et al. (2019) investigated the effect of a video game-based exercise programme against a control group for 8 weeks on fear of movement/re-injury. The results showed that there were no significant between group difference in fear of movement/re-injury (β = −2.97, 95% CI = −6.14 to 0.21, *p* =0 0.07) post-intervention at 8 weeks. Cana-Pino et al. (2023) examined the effect of two exercise modalities: Supervised Exercise (SE)/Laser Guided Exercise (LGE) and Pain Neuroscience Education (PNE) on fear of movement/kinesiophobia in participants with Non-Specific Chronic Low Back Pain (NSCLBP). The result showed a significant between-group difference post-intervention scores at 20 weeks in terms of kinesiophobia (TSK-11) (*p* < 0.05) and a high effect size (d = 0.81). Another study (Ogunniran et al., 2023) examined the effects of Kinesiology taping (KT) and Core-stabilization exercises (CSE) separately and in combination on Kinesiophobia in patients with NSCLBP. The results showed that there was statistically significant difference in kinesiophobia (*p* < 0.001) across the three groups at the end of 8 week post-intervention. KT and CSE showed the greatest improvement in kinesiophobia at 8 weeks (21.08 ± 3.75 95% CI: 18.70 to 23.47), CSE (34.79 ± 7.89 95% CI: 29.81 to 39.73) and KT (36.40 ± 8.40 95% CI: 30.39 to 42.41) compared to baseline KT and CSE (42.54 ± 7.37 95% CI: 37.47 to47.20), CSE (42.24 ± 7.64 95% CI: 37.98 to 48.33) and KT (41.31 ± 9.10 95% CI: 34.32 to 46.28).

### Chronic thoracic pain

There were no studies that specifically investigated people with chronic thoracic pain. However, a study by Galan-Martin et al. (2020) investigated the effect of an 11-week physical exercise programme (combined with education) on people with chronic spinal (cervical, thoracic, LBP, and combined) pain and found a significant improvement in kinesiophobia levels in the exercise group at 6 months when compared to baseline scores and the control group.

## Discussion

This systematic review is the first to investigate and compare the effect of different exercise/physical activity interventions in reducing kinesiophobia in people with chronic non-specific spine-related pain. The review indicated that there are a variety of exercises that are effective in reducing kinesiophobia in people with chronic spine-related pain, but that the majority of studies had been conducted on people with chronic non-specific LBP. In contrast only three studies investigated neck pain, and none focused specifically on thoracic pain, highlighting the need for further research to investigate the effects of exercise on kinesiophobia in people with chronic non-specific neck and thoracic pain. The exercises with significant effect in reducing kinesiophobia in people with chronic LBP were Pilates (multiple types), water-based stretching and strengthening. Miyamoto et al. (2018) and Cruz-Díaz et al. (2018) support the use of Pilates to reduce kinesiophobia in people with LBP, but the studies have methodological issues that raise concerns of bias which affects the reliability of the findings. Miyamoto et al. (2018) reported that they were unable to blind participants to the intervention groups, which may have affected the performance of participants (Karanicolas et al., 2010). Additionally, Cruz-Díaz et al. (2018) reported that there were significant differences in body mass index between the two groups prior to intervention.

The studies by Cruz-Díaz et al. (2017) and Da Luz Junior et al. (2014) were deemed as having low risk of bias. Both studies show a beneficial direction of effect in reducing kinesiophobia following mat and equipment-based Pilates. Additionally, Da Luz Junior et al. (2014) concluded that equipment-based Pilates has a longer lasting effect in reducing kinesiophobia. The proportion of males to females in both studies were not proportionate, with females outweighing males, which may compromise generalisability of these findings. The imbalance of genders is a limitation in both studies and future studies should aim to recruit an equal number of men and women. There has been some indication that women with chronic non-specific LBP have greater levels of kinesiophobia than men on movements or actions that require dynamic balance (Kahraman et al., 2018), and this difference indicates that both genders should be equally investigated, to compare any potential differences in kinesiophobia and the effects exercise may have.

The notion that exercise has a beneficial effect on kinesiophobia in people with chronic spine-related pain is evidenced by the findings of Galan-Martin et al. (2020), who found kinesiophobia was reduced more in those who underwent physical exercise than those having physiotherapy treatment excluding exercise. This study was the only one to have participants who had chronic non-specific pain from any area in the spine or more than one area. This study was deemed high risk because of incomplete blinding; both the participant and Physiotherapist (who led the session) were not blinded. Findings from Miyamoto et al. (2013), shows that exercise/Pilates combined with education was not significantly different to education alone in reducing kinesiophobia. On the other hand, supervised exercise with or without laser guided exercise, when combined with PNE, reduces kinesiophobia in patients with NSCLBP (Cana-Pino et al., 2023). However, the effect of exercise on kinesiophobia cannot be ascertained since it has been performed in combination with PNE and the effect was only assessed for 3 months, longer term effect cannot be showed. Findings from Ogunniran et al. (2023) shows improvement in Kinesiophobia in the three groups (KT and CSE group, CSE only group and KT only group). Although, improvement was greater in the combined KT and CSE group, showing that modalities such as KT and CSE are effective in the treatment of NSCLBP. Additionally, a study by Hernandez-Lucas et al. (2023) evaluating the effects of the Back School on non-specific neck pain in adults aged ≥18, showed a beneficial effect on kinesiophobia, however, this study did not have a post-intervention follow-up, making it difficult to ascertain its long-term effect. Furthermore, due to the small sample size, generalisability of the results may be compromised. Conversely, (Martins de Sousa et al., 2022) reported that high/low frequency TENS, compared to placebo TENS, in combination with exercise did not provide clinical benefits to patients with chronic neck pain.

Overall, the risk of bias was moderate/high in ~80% of the included studies. Based on the GRADE assessment, there is only very low certainty of evidence that exercise/physical activity are effective at reducing fear of movement, primarily due to the wide variations between the included studies in terms of exercise/physical activity modalities and outcome assessment tools. A systematic review and meta-analysis by Hanel et al. (2020) had similar findings to this review; they found that exercise improved kinesiophobia in patients with chronic LBP, but the overall quality of evidence was “very low to low”.

### Strengths and limitations

The current review has several strengths. The review protocol was registered in PROSPERO, published (Jadhakhan et al., 2022) and was conducted and reported in accordance with the PRISMA 2020 statement (Page et al., 2021). Two independent reviewers were involved in study selection, data extraction and quality assessment, and a third reviewer was available to ensure overall methodological consistency and to resolve any disagreements. To ensure an exhaustive review of the available literature, a comprehensive search strategy was implemented with broad inclusion criteria. However, there are some limitations which should be noted. Significant heterogeneity was found between the included studies, particularly pertaining to methods of ascertaining exercise/physical activity, variation in chronic non-specific spine-related pain and measures used to assess kinesiophobia which precluded meta-analysis. Furthermore, due to limited data on ethnicity and gender, sub-group analyses to explore the effect of these variable was not possible. The generalisability and applicability of these findings may be reduced, because of the setting, population and criteria to ascertain kinesiophobia. Additionally, there were limitations in the study selection process such as the search was restricted to English language publications.

## Conclusion

Exercise/physical activity can significantly reduce kinesiophobia in people with chronic non-specific spine-related pain, although the majority of the studies included in this review investigated chronic non-specific LBP. Favorable short-term effects on kinesiophobia were evidenced from Pilates (equipment-based) exercises for people with LBP. General exercise and strengthening training appeared to also be effective at reducing kinesiophobia in people with LBP. However, the overall the certainty of the evidence was very low. Further research should consider exploring the effect exercise has on kinesiophobia in people with chronic non-specific spine-related pain, with robust methodology required to produce the highest quality of evidence. Additionally, further research is needed to investigate the effect exercise has on kinesiophobia for people with chronic non-specific neck and thoracic pain, as there was very limited evidence to draw any meaningful conclusions.

## Data availability statement

The original contributions presented in the study are included in the article/Supplementary material, further inquiries can be directed to the corresponding author.

## Author contributions

DF and FJ conceived the study design. RS drafted the first version of this review and this was reviewed and revised by DF and FJ. The search strategy was developed by RS and FJ and iteration discussed with DF. The search was performed by RS. FJ and RS performed screening for study selection, collected data from the included studies, conducted quality assessment, and performed data analysis/synthesis. DF is guarantor. All authors contributed to the article and approved the submitted version.

## References

[B1] Akers J. Aguiar-Ibáñez R. Sari A. B. A. Beynon S. Booth A. Burch J. . (2009). Systematic Reviews: CRD's Guidance for Undertaking Reviews in Health Care. New York, NY: Centre for Reviews and Dissemination, University of York.

[B2] Akodu A. K. Nwanne C. A. Fapojuwo O. A. (2021). Efficacy of neck stabilization and Pilates exercises on pain, sleep disturbance and kinesiophobia in patients with non-specific chronic neck pain: a randomized controlled trial. J. Bodywork Movement Therap. 26, 411–419. 10.1016/j.jbmt.2020.09.008 33992276

[B3] Balci N. Ç. Aytar A. Atici E. Taşkin G. Gülşen M. Demirsoz M. . (2020). The effect of aquatic and land exercises on pain, health related quality of life, kinesiophobia and disability in chronic low back pain: a randomized clinical trial. J. Novel Physiother. Phys. Rehab. 7, 062–067. 10.17352/2455-5487.000082

[B4] Balshem H. Helfand M. Schünemann H. J. Oxman A. D. Kunz R. Brozek J. . (2011). GRADE guidelines: 3. Rating the quality of evidence. J. Clin. Epidemiol. 64, 401–406. 10.1016/j.jclinepi.2010.07.015 21208779

[B5] Bevan S. (2012). The Impact of Back Pain on Sickness Absence in Europe. Available online at: https://www.google.com/url?sa=tandrct=jandq=andesrc=sandsource=webandcd=andved=2ahUKEwjPsOSwn9_3AhVSdcAKHTP5BgAQFnoECAgQAwandurl=https%3A%2F%2F%2Fbritishlibrary%2F~%2Fmedia%2Fbl%2Fglobal%2Fbusiness-and-management%2Fpdfs%2Fnon-secure%2Fi%2Fm%2Fp%2Fimpact-of-back-pain-on-sickness-absence-in-europe.pdfandusg=AOvVaw1QjAnUknpBssOjHygZSqPQ (accessed May 14, 2022).

[B6] Bordeleau M. Vincenot M. Lefevre S. Duport A. Seggio L. Breton T. . (2022). Treatments for kinesiophobia in people with chronic pain: A scoping review. Fron. Behav. Neurosci. 20, 1–18. 10.3389/fnbeh.2022.933483 36204486 PMC9531655

[B7] Cana-Pino A. Apolo-Arenas M. D. Falla D. Lluch-Girbes E. Espejo-Antúnez L. (2023). Supervised exercise with or without laser-guided feedback for people with non-specific chronic low back pain. A randomized controlled clinical trial. J. Electro. Kinesiol. 70, 1–9. 10.1016/j.jelekin.2023.102776 37163815

[B8] Caspersen C. J. Powell K. E. Christenson G. M. (1985). Physical activity, exercise, and physical fitness: definitions and distinctions for health-related research. Public Health Rep. 100, 126–131. 3920711 PMC1424733

[B9] Cruz-Díaz D. Bergamin M. Gobbo S. Martínez-Amat A. Hita-Contreras F. (2017). Comparative effects of 12 weeks of equipment based and mat pilates in patients with chronic low back pain on pain, function and transversus abdominis activation: a randomized controlled trial. Compl. Ther. Med. 33, 72–77. 10.1016/j.ctim.2017.06.004 28735829

[B10] Cruz-Díaz D. Romeu M. Velasco-González C. Martínez-Amat A. Hita-Contreras F. (2018). The effectiveness of 12weeks of Pilates intervention on disability, pain and kinesiophobia in patients with chronic low back pain: a randomized controlled trial. Clin. Rehab. 32, 1249–1257. 10.1177/0269215518768393 29651872

[B11] Da Luz Junior M. A. Pena Costa L. O. Fuhro F. F. Manzoni A. C. T. Oliveira N. T. B. Cabral C. M. N. (2014). Effectiveness of mat Pilates or equipment-based Pilates exercises in patients with chronic nonspecific low back pain: a randomized controlled trial. Physical Therapy. 94, 623–31. 10.2522/ptj.20130277 24435105

[B12] De Freitas D. Costa C. D. Civile V. T. (2020). Effects of the pilates method on kinesiophobia associated with chronic non-specific low back pain: systematic review and meta-analysis. J. Bodywork Movem. Ther. 24, 300–306. 10.1016/j.jbmt.2020.05.005 32826004

[B13] De Moraes V. de Góes Salvetti É. B. Damiani M. L. P. de Mattos Pimenta C. A. (2014). Self-efficacy and fear avoidance beliefs in chronic low back pain patients: coexistence and associated factors. Pain Manage. Nurs. 15, 593–602. 10.1016/j.pmn.2013.04.004 23891180

[B14] Galan-Martin M. A. Montero-Cuadrado F. Lluch-Girbes E. Coca-López M. C. Mayo-Iscar A. Cuesta-Vargas A. . (2020). Pain neuroscience education and physical therapeutic exercise for patients with chronic spinal pain in spanish physiotherapy primary care: a pragmatic randomized controlled trial. J. Clin. Med. 9 1201. 10.3390/jcm9041201 32331323 PMC7230486

[B15] Guyatt G. Oxman A. D. Akl E. A. Kunz R. Vist G. Brozek J. . (2011). GRADE guidelines: 1. Introduction-GRADE evidence profiles and summary of findings tables. J. Clin. Epidemiol. 64 383–394. 10.1016/j.jclinepi.2010.04.026 21195583

[B16] Hanel J. Owen P. J. Held S. Tagliaferri S. D. Miller C. T. Donath L. . (2020). Effects of exercise training on fear-avoidance in pain and pain-free populations: systematic review and meta-analysis. Sports Med. 50, 2193–2207. 10.1007/s40279-020-01345-1 32946074

[B17] Hernandez-Lucas P. Leirós-Rodríguez R. Lopez-Barreiro J. García-Soidán J. L. (2023). Effects of back school-based intervention on non-specific neck pain in adults: a randomized controlled trial. BMC Sports Sci. Med. Rehab. 15, 4–10. 10.1186/s13102-023-00666-8 37069599 PMC10111684

[B18] Hey H. W. D. Lim J. X. Y. Ong J. Z. Luo N. (2021). epidemiology of neck pain and its impact on quality-of-life—a population-based, cross sectional study in Singapore. Spine, 46, 1572–1580. 10.1097/BRS.0000000000004071 34714794

[B19] Higgins J. P. T. Thomas J. Chandler J. Cumpston M. Li T. Page M. J. . (2021). Cochrane Handbook for Systematic Reviews of Interventions Version 6, 3. (updated February 2022). Available online at: www.training.cochrane.org/handbook (accessed July 6, 2023)

[B20] Hogg-Johnson S. van der Velde G. Carroll L. J. Holm L. W. Cassidy J. D. Guzman J. . (2009). The burden and determinants of neck pain in the general population: results of the bone and joint decade 2000–2010 task force on neck pain and its associated disorders. J. Manip. Physiol. Ther. 32, S46–S60. 10.1016/j.jmpt.2008.11.010 19251074

[B21] Hudes K. (2011). The tampa scale of kinesiophobia and neck pain, disability and range of motion: a narrative review of the literature. The J. Can. Chiropr. Assoc. 55, 222–232. 21886284 PMC3154068

[B22] Jadhakhan F. Sobeih R. Falla D. (2022). Effects of exercise/physical activity on fear of movement in people with spine-related pain: protocol for a systematic review and meta-analysis. BMJ Open 12, e060264. 10.1136/bmjopen-2021-060264 35589367 PMC9121489

[B23] Kahraman B. O. Kahraman T. Kalemci O. Sengul Y. S. (2018). Gender differences in postural control in people with nonspecific chronic low back pain. Gait Posture 64, 147–151. 10.1016/j.gaitpost.2018.06.026 29909228

[B24] Karanicolas P. J. Farrokhyar F. Bhandari M. (2010). Practical tips for surgical research: blinding: who, what, when, why, how? Can. J. Surg. 53, 345–348. 20858381 PMC2947122

[B25] Keane L. G. (2017). Comparing aquastretch with supervised land based stretching for chronic lower back pain. J. Bodywork Movem. Ther. 21, 297–305. 10.1016/j.jbmt.2016.07.004 28532872

[B26] Leeuw M. Vlaeyen J. W. S. Crombez G. (2007). *Disability, Fear of Movement*. Encyclopedia of Pain. Cham: Springer.

[B27] Liu H. Huang L. Yang Z. Li H. Wang Z. Peng L. . (2021). Fear of movement/(Re) injury: an update to descriptive review of the related measures. Front. Psychol. 12, 696762. 10.3389/fpsyg.2021.696762 34305755 PMC8292789

[B28] Luque-Suarez A. Martinez-Calderon J. Falla D. (2019). Role of kinesiophobia on pain, disability and quality of life in people suffering from chronic musculoskeletal pain: a systematic review. Br. J. Sports Med. 53, 554–559. 10.1136/bjsports-2017-098673 29666064

[B29] Martins de Sousa P. H. Fidelis-de-Paula-Gomes C. A. Pontes-Silva A. Milena Fernanda Pereira H. Gardhel Costa Araujo G. Eduardo Kalatakis-dos-Santos A. (2022). Additional effect of transcutaneous electrical nerve stimulation in a therapeutic exercise program for sedentary with chronic neck pain: A double-blind randomized controlled trial. Physiother. Res. Int. 28, 25–30. 10.1002/pri.1978 36252091

[B30] Miller R. P. Kori S. H. Todd D. D. (1991). The tampa scale: a measure of kinisophobia. Clin. J. Pain 7 51. 10.1097/00002508-199103000-00053

[B31] Miyamoto G. C. Costa L. O. P. Galvanin T. Cabral C. M. N. (2013). Efficacy of the addition of modified pilates exercises to a minimal intervention in patients with chronic low back pain: a randomized controlled trial. Phys. Ther. 93, 310–320. 10.2522/ptj.20120190 23064732

[B32] Miyamoto G. C. Franco K. F. M. van Dongen J. M. Franco Y. R. D. S. Oliveira D. Amaral N. T. B. . (2018). Different doses of Pilates-based exercise therapy for chronic low back pain: a randomised controlled trial with economic evaluation. Br. J. Sports Med. 52, 859–868. 10.1136/bjsports-2017-098825 29525763

[B33] Nassif H. Brosset N. Guillaume M. Delore-Milles E. Tafflet M. Buchholz F. J.F. . (2011). Evaluation of a randomized controlled trial in the management of chronic lower back pain in a french automotive industry: an observational study. Arch. Phys. Med. Rehab. 92, 1927–1936. 10.1016/j.apmr.2011.06.029 22133239

[B34] National Institute for Health Care Excellence (2022). Back Pain – Low (Without Radiculopathy): How Common is It?. Available online at: https://cks.nice.org.uk/topics/back-pain-low-without-radiculopathy/background-information/prevalence/ (accessed May 12, 2022).

[B35] Ogunniran I. A. Akodu A. K. Odebiyi D. O. (2023). Effects of kinesiology taping and core stability exercise on clinical variables in patients with non-specific chronic low back pain: a randomized controlled trial. J. Bodywork Movem. Ther. 33, 20–27. 10.1016/j.jbmt.2022.09.013 36775519

[B36] Page M. J. McKenzie J. E. Bossuyt P. M. Boutron I. Hoffmann T. C. Mulrow C. D. . (2021). The PRISMA 2020 statement: an updated guideline for reporting systematic reviews. BMJ 372, n71. 10.1136/bmj.n71 33782057 PMC8005924

[B37] Richardson W. S. Wilson M. C. Nishikawa J. Hayward R. S. (1995). The well-built clinical question: a key to evidence-based decisions. ACP J. Club 123, A12–A13. 10.7326/ACPJC-1995-123-3-A12 7582737

[B38] Safiri S. Kolahi A. A. Hoy D. Buchbinder R. Mansournia M. A. Bettampadi D. . (2020). Global, regional, and national burden of neck pain in the general population, 1990-2017: systematic analysis of the global burden of disease study 2017. BMJ 368, m791. 10.1136/bmj.m791 32217608 PMC7249252

[B39] Schünemann H. J. Higgins J. P. T. Vist G. E. Glasziou P. Akl E. A. Skoetz N. . (2021). Cochrane Handbook for Systematic Reviews of Interventions: The Cochrane Collaboration; 2021. Available online at: https://training.cochrane.org/handbook/current (accessed April 27, 2023).

[B40] Sharma S. Ferreira-Valente A. Williams C. Abbott A. C. Pais-Ribeiro J. H. J. Jensen M. P. (2020). Group differences between countries and between languages in pain-related beliefs, coping, and catastrophizing in chronic pain: a systematic review. Pain Med. 21, 1847–1862. 10.1093/pm/pnz373 32044980 PMC7553014

[B41] Sterne J. A. Savović J. Page M. J. Elbers R. G. Blencowe N. S. Boutron I. . (2019). RoB 2: a revised tool for assessing risk of bias in randomised trials. BMJ 366, l4898. 10.1136/bmj.l4898 31462531

[B42] Tagliaferri S. D. Miller C. T. Ford J. J. Hahne A. J. Main L. C. Rantalainen T. . (2020). Randomized trial of general strength and conditioning versus motor control and manual therapy for chronic low back pain on physical and self-report outcomes. J. Clin. Med. 9, 1726. 10.3390/jcm9061726 32503243 PMC7355598

[B43] Trocoli T. O. Botelho R. V. (2016). Prevalence of anxiety, depression and kinesiophobia in patients with low back pain and their association with the symptoms of low back spinal pain. Rev. Bras. Reumatol. 56, 330–336. 10.1016/j.rbr.2015.09.009 27476626

[B44] Varallo G. Giusti E. M. Scarpina F. Cattivelli R. Capodaglio P. Castelnuovo G. . (2020). The association of kinesiophobia and pain catastrophizing with pain-related disability and pain intensity in obesity and chronic lower-back pain. Brain Sci. 11, 11. 10.3390/brainsci11010011 33374178 PMC7823580

[B45] Vicente-Campos D. Sanchez-Jorge S. Terrón-Manrique P. Guisard M. Collin M. Castaño B. . (2021). The main role of diaphragm muscle as a mechanism of hypopressive abdominal gymnastics to improve non-specific chronic low back pain: a randomized controlled trial. J. Clin. Med. 10 4983. 10.3390/jcm10214983 34768502 PMC8584898

[B46] Vincent H. K. George S. Z. Seay A. N. Vincent K. R. Hurley R. W. (2014). Resistance exercise, disability, and pain catastrophizing in obese adults with back pain. Med. Sci. Sports Exerc. 46, 1693–701. 10.1249/MSS.0000000000000294 25133997 PMC4137474

[B47] Vlaeyen J. W. S. Kole-Snijders A. M. J. Rotteveel A. M. Ruesink R. Heuts P. H. T. G. (1995). The role of fear of movement/(re) injury in pain disability. J. Occup. Rehab. 5, 235–252. 10.1007/BF02109988 24234727

[B48] Vlaeyen J. W. S. Linton S. J. (2000). Fear-avoidance and its consequences in chronic musculoskeletal pain: a state of the art. Pain 85, 317–332. 10.1016/S0304-3959(99)00242-0 10781906

[B49] Vos T. (2020). Global burden of 369 diseases and injuries in 204 countries and territories, 1990–2019: a systematic analysis for the global burden of disease study 2019. The Lancet 396, 1204–1222. 10.1016/S0140-6736(20)30925-9 33069326 PMC7567026

[B50] Waddell G. Newton M. Henderson I. Somerville D. Main C. J. (1993). A fear-avoidance beliefs questionnaire (FABQ) and the role of fear-avoidance beliefs in chronic low back pain and disability. Pain 52, 157–168. 10.1016/0304-3959(93)90127-B 8455963

[B51] World Health Organisation (2021). Ageing and Health. Available online at: https://www.who.int/news-room/fact-sheets/detail/ageing-and-health (accessed March 30, 2022).

[B52] Zadro J. R. Shirley D. Simic M. Mousavi S. J. Ceprnja D. Maka K. . (2019). Video-game-based exercises for older people with chronic low back pain: a randomized controlledtable trial (GAMEBACK). Phys. Ther. 99, 14–27. 10.1093/ptj/pzy112 30247715

