# Supplementary material for: Effects of exercise/physical activity on fear of movement in people with spine-related pain: a systematic review
Source: Front Psychol. 2023 Jul 27;14:1213199. doi: 10.3389/fpsyg.2023.1213199 (PMC10415102; doi:10.3389/fpsyg.2023.1213199)
Supplement: Supplementary file 1 [file Data_Sheet_1.pdf]

Supplementary material 1: Exercise, pain, fear of movement and randomised controlled trials search Strategy

Search from inception to 22nd June 2023

Medline search strategy

Exercis*.mp **OR** Training **OR** exercise training.mp **OR** Motor control.mp **OR** physical activit*.mp **OR** aerobic exercise.mp **OR** physical exercise.mp **OR** resistance training.mp **OR** strength training.mp **OR** endurance exercise.mp **OR** cardiovascular.mp **OR** exercise.mp

AND

Pain.mp **OR** Non specific.mp **OR** Spinal pain.mp **OR** Chronic non specific pain.mp **OR** Mechanical pain.mp **OR** Spine related pain.mp **OR** Neck pain.mp **OR** Thoracic pain.mp **OR** Low back pain.mp **OR** back pain

AND/OR

Kinesiophobia.mp **OR** Fear of movement.mp **OR** Tampa scale.mp **OR** TSK* **OR** Fear avoidance behavi* questionnaire.mp **OR** FABQ*

AND

Randomi* controlled trial.mp **OR** Non-randomi* controlled trial.mp **OR** Control trial.mp **OR** Intervention.mp **OR** Clinical trial*.mp **OR** comparison group.mp **OR** control group.mp **OR** non-random.mp

CINAHL search strategy

Exercise **OR** Training **OR** exercise training **OR** Motor control **OR** physical activity **OR** aerobic exercise **OR** physical exercise **OR** resistance **t**raining **OR** strength training **OR** endurance exercise **OR** cardiovascular **OR** exercise

AND

Pain **OR** Nonspecific **OR** Spinal pain **OR** Chronic nonspecific pain **OR** Mechanical pain **OR** Spine related pain **OR** Neck pain **OR** Thoracic pain **OR** Low back pain **OR** back pain

AND/OR

Kinesiophobia **OR** Fear of movement **OR** Tampa scale **OR** TSK **OR** Fear avoidance behaviour questionnaire **OR** FABQ

AND

Randomised controlled trial **OR** Nonrandomised controlled trial **OR** Control trial **OR** Intervention **OR** Clinical trial **OR** comparison group **OR** control group **OR** non-random

EMBASE search strategy

Exercis*.mp **OR** Training **OR** exercise training.mp **OR** Motor control.mp **OR** physical activit*.mp **OR** aerobic exercise.mp **OR** physical exercise.mp **OR** resistance training.mp **OR**

strength training.mp **OR** endurance exercise.mp **OR** cardiovascular.mp **OR** exercise.mp **OR** Exercise.tw **OR** exercise.mp **OR** physical activity.mp

AND

Pain.mp **OR** Non specific.mp **OR** Spinal pain.mp **OR** Chronic non specific pain.mp **OR** Mechanical pain.mp **OR** Spine related pain.mp **OR** Neck pain.mp **OR** Thoracic pain.mp **OR** Low back pain.mp **OR** back pain

AND/OR

Kinesiophobia.mp **OR** Fear of movement.mp **OR** Tampa scale.mp **OR** TSK* **OR** Fear avoidance behavi* questionnaire.mp **OR** FABQ* **OR** kinesiophobia.mp **OR** fear of movement.mp

AND

Randomi* controlled trial.mp **OR** Nonrandomi* controlled trial.mp **OR** Control trial.mp **OR** Intervention.mp **OR** Clinical trial*.mp **OR** comparison group.mp **OR** control group.mp **OR** nonrandom.mp **OR** randomi* controlled trial.mp

risk.mp. [mp/ = indexing term (Emtree heading).

exp = exploded indexing term (Emtree heading).

$ = truncation.

ti,ab = terms in either title or abstract fields.

=title, abstract
